# Phase II study of gemcitabine and vindesine in patients with previously untreated non-resectable non-small-cell lung cancer

**DOI:** 10.1038/sj.bjc.6690140

**Published:** 1999-02

**Authors:** J B Sørensen, B Bergman, A L Nielsen, M Krarup, P Dombernowsky, H H Hansen

**Affiliations:** Department of Oncology, Finsen Center, National University Hospital, Copenhagen, Denmark; Department of Respiratory Medicine, Sahlgrenska University Hospital, Gothenburg, Sweden; Department of Oncology, Herlev Hospital, Copenhagen, Denmark

**Keywords:** non-small cell lung cancer, chemotherapy, gemcitabine, vindesine

## Abstract

Because both vindesine and gemcitabine are active drugs in advanced non-small-cell lung cancer (NSCLC), with different modes of action and only partly overlapping toxicity, a phase II study was performed. Gemcitabine 1000 mg m^−2^ was given on days 1, 8 and 15 every 4 weeks, while vindesine 3 mg m^−2^ was administered weekly for 7 weeks, then every 2 weeks. A total of 42 patients with nonresectable NSCLC were included. The median age of patients was 56 years; 57% were men, 52% had adenocarcinoma, 31% squamous cell carcinoma and 17% had large-cell carcinoma. The performance status ranged from 0 to 2 with 83% in performance status 1. The majority (55%) had stage IV disease, while 40% had stage III B and 5% stage III A disease. WHO grade 3–4 leucopenia occurred in five patients (12%) and 9% had grade 4 neutropenia. Thrombocytopenia grade 3–4 was observed in six patients (15%). There were no septic death or bleeding episodes. One patient had a transient WHO grade 4 increase in bilirubin, and four patients had a decrease in glomerular filtration rate below the normal limit; one of these patients developed a non-reversible renal insufficiency. Ten patients (24%) complained of dyspnoea of uncertain mechanism, possibly involving bronchoconstriction. There were one complete and seven partial responses among 40 assessable patients (20%, 95% confidence limits 9–36%). Median response duration was 31 weeks (range 11–83 weeks) and median survival time 31 weeks (range 2–171 weeks). The current combination of gemcitabine and vindesine does not appear to be promising for further examination because of the toxicity and somewhat disappointing activity. © 1999 Cancer Research Campaign


*British Journal of Cancer* (1999) **79**(5/6), 875–881
© 1999 Cancer Research Campaign
Article no. bjoc.1998.0140

### Phase II study of gemcitabine and vindesine in patients with previously untreated non-resectable non-small-cell lung cancer

**JB Sørensen[1], B Bergman[2], AL Nielsen[3], M Krarup[1], P Dombernowsky[3] and HH Hansen[1]**

[1]Department of Oncology, Finsen Center, National University Hospital, Copenhagen, Denmark; [2]Department of Respiratory Medicine, Sahlgrenska University Hospital, Gothenburg, Sweden; [3]Department of Oncology, Herlev Hospital, Copenhagen, Denmark

**Summary** Because both vindesine and gemcitabine are active drugs in advanced non-small-cell lung cancer (NSCLC), with different modes of action and only partly overlapping toxicity, a phase II study was performed. Gemcitabine 1000 mg $m^{-2}$ was given on days 1, 8 and 15 every 4 weeks, while vindesine 3 mg $m^{-2}$ was administered weekly for 7 weeks, then every 2 weeks. A total of 42 patients with nonresectable NSCLC were included. The median age of patients was 56 years; 57% were men, 52% had adenocarcinoma, 31% squamous cell carcinoma and 17% had large-cell carcinoma. The performance status ranged from 0 to 2 with 83% in performance status 1. The majority (55%) had stage IV disease, while 40% had stage III B and 5% stage III A disease. WHO grade 3–4 leucopenia occurred in five patients (12%) and 9% had grade 4 neutropenia. Thrombocytopenia grade 3–4 was observed in six patients (15%). There were no septic death or bleeding episodes. One patient had a transient WHO grade 4 increase in bilirubin, and four patients had a decrease in glomerular filtration rate below the normal limit; one of these patients developed a non-reversible renal insufficiency. Ten patients (24%) complained of dyspnoea of uncertain mechanism, possibly involving bronchoconstriction. There were one complete and seven partial responses among 40 assessable patients (20%, 95% confidence limits 9–36%). Median response duration was 31 weeks (range 11–83 weeks) and median survival time 31 weeks (range 2–171 weeks). The current combination of gemcitabine and vindesine does not appear to be promising for further examination because of the toxicity and somewhat disappointing activity.

**Key words:** non-small cell lung cancer; chemotherapy; gemcitabine, vindesine

Although chemotherapy in non-resectable non-small cell lung cancer (NSCLC) leads to a modest survival increase (Souquet et al, 1993), the effect in this disease is far from satisfactory (Sørensen et al, 1994). Median survival in patients with advanced NSCLC receiving chemotherapy varies generally from 5 to 9 months, with less than 5% surviving 5 years (Comis et al, 1995). More effective chemotherapy is needed and development of new active regimens is necessary to achieve this goal.

Although chemotherapy in NSCLC is considered to be palliative, the effects of treatment on patients' quality of life are unclear. Statements on palliative effects from treatment often pertain to physicians' ratings of performance status (Bakker et al, 1986; Ganz et al, 1989; Minet et al, 1987), although conclusive data on quality of life based upon patient self-ratings are sparse (Bartel-Copel et al, 1997). Nevertheless, several recent publications and reports on NSCLC have incorporated quality-of-life assessment in the treatment evaluation (Mastekaasa, 1988; Maasilta et al, 1990; Sarna, 1990; Kosty et al, 1994; Pujol et al, 1994; Billingham et al, 1997; Helsing et al, 1998), indicating an increasing awareness of one of the primary therapeutic goals with currently available regimens, i.e. palliation.

*Received 29 September 1997*
*Revised 18 May 1998*
*Accepted 4 June 1998*

*Correspondence to:* JB Sørensen, Department of Oncology, Finsen Center, Copenhagen University Hospital/Rigshospitalet, 9 Blegdamsvej, DK 2100 Copenhagen, Denmark

Vindesine is one of the active drugs used in NSCLC treatment with an accumulated response rate of 18% based on single-agent treatment of 295 patients included in phase II trials (95% confidence limits 13–22%) (Sørensen et al, 1993). The maximal tolerated dose when used as single agent is 4 mg $m^{-2}$ i.v. weekly for 5–10 weeks followed by biweekly administration, but the majority of studies have used 3 mg $m^{-2}$ (Sørensen et al, 1993). The antineoplastic effect is exerted through binding to tubulin units, which prevents ordered assembly into microtubules and, instead, at low concentrations leads to the formation of pseudo-crystaline structures that do not appear to have any functional activity. The acute dose-limiting toxicity is myelosuppression, with a nadir at days 7 or 8 and recovery by days 11–13, while neurotoxicity is the limiting factor in more prolonged treatment (Bayssas et al, 1980).

Gemcitabine (2′,2′-difluorodeoxycytidine) is a deoxycytidine analogue with structural and metabolic similarities to cytarabine. In contrast with cytarabine, gemcitabine has shown activity in a variety of solid tumours, including NSCLC. Gemcitabine is phosphorylated intracellularly, and in this form inhibits DNA synthesis through inhibition of DNA polymerase (Guchelarr et al, 1996). Several schedules for administration of gemcitabine have been explored, and a weekly schedule three times every 4 weeks appears to be the most favourable (Guchelaar et al, 1996). The side-effects are schedule dependent, consisting of haematological toxicity, flu-like syndrome, fever, hypotension and liver toxicity. The flu-like syndrome is dose limiting with frequent administration, such as treatment for 5 consecutive days every 3 weeks, whereas myelosuppression with thrombocytopenia (more important than granulocytopenia) and liver toxicity are dose limiting when gemcitabine is administered at longer intervals, such as weekly. Less common side-effects are skin rash, dyspnoea, oedema and proteinuria, mild alopecia, nausea and occasional vomiting (Guchelaar et al, 1996). Gemcitabine has, in five studies, involving a total of 546 patients evaluated three times weekly every 4 weeks, revealed a 20% response rate (95% confidence limits 17–23%; Hansen and Sørensen, 1997). Doses have ranged from 800 to 2100 mg $m^{-2}$ weekly.

The documented activity of gemcitabine and vindesine when used as single agents, together with their different modes of action, combining a pyrimidine antagonist with a tubulin inhibitor, make a combination of gemcitabine and vindesine attractive for evaluation in patients with non-respectable NSCLC, with respect to both antineoplastic effect and impact on quality of life.

### PATIENTS AND METHODS

### Patients

The study was conducted according to the Declaration of Helsinki, with the protocol being approved by the local ethics committees. Patients with non-respectable NSCLC stage III A, III B or IV, aged 18–70 years entered the study after giving written informed consent. Other criteria for study entry included no prior chemotherapy, bidimensionally or unidimensionally measurable disease, World Health Organization (WHO) performance status 0–2, life expectancy 12+ weeks, no radio- or corticosteroid therapy within the previous 3 weeks, leucocytes $\geq 3.0 \times 10^9$ $l^{-1}$, platelets $\geq 100 \times 10^9$ and haemoglobin $\geq$ 6.7 mmol $l^{-1}$. Exclusion criteria were active infection, brain metastasis, hypercalcaemia, second malignancy, creatinine level greater than 0.15 mmol $l^{-1}$, serum bilirubin level greater than twice the upper normal limit, aspartate transaminases > 3 times normal and prothrombin > 1.5 times normal. Also, patients with peripheral neuropathy or significant neurological or psychiatric disease were excluded. Bone metastasis could not be used as the sole indicator lesion.

Pretherapy evaluation included a medical examination, WHO performance score, complete blood cell count, biochemical profile, liver function tests, ECG, chest radiographs and urine analysis. A computerized tomographic scan was performed if disease was not measurable clinically or on chest radiography. Renal function was evaluated by chrom-EDTA clearance every 8 weeks (Groth et al, 1981).

Before and after each chemotherapy course, patients' vital signs and temperature were recorded. The routine blood tests (complete blood count, biochemical profile, liver function tests) and urine analysis were repeated weekly. Chest radiography and other examinations necessary for evaluating response status were performed every 4 weeks.

### Treatment

Gemcitabine 1000 mg $m^{-2}$ was dissolved in 0.9% saline to a concentration of 20 mg $ml^{-1}$ or less and infused over 30 min on days 1, 8 and 15, with no gemcitabine being administered on day 22. Courses were repeated every 28 days. Vindesine 3 mg $m^{-2}$ diluted to a concentration of 1 mg $m^{-2}$ was administered intravenously over 10 min weekly for 7 weeks, then every two weeks. Treatment with gemcitabine and vindesine for 28 days constituted one treatment course.

### Response to therapy

WHO criteria were used in response assessment and evaluation of toxicity (WHO, 1979). The duration of response was calculated from the first day of chemotherapy until disease progression or death.

In unidimensionally measurable lesions, partial remission was defined as a definite decrease in the size of lesions of at least 50% as evaluated by two observers and with a duration of at least 4 weeks. Treatment was discontinued in the case of disease progression or if severe toxicity was observed. Patients with disease response or stable disease continued therapy until progression.

### Dose modifications

The dose of both gemcitabine and vindesine was reduced to 75% if the white blood cell count was $2.0–2.9 \times 10^9$ $l^{-1}$ or the thrombocytes were $50–99 \times 10^9$ $l^{-1}$ when chemotherapy was due. Both gemcitabine and vindesine were omitted if white blood cell count was less than $2.0 \times 10^9$ $l^{-1}$ or the thrombocyte count was less than $50 \times 10^9$ $l^{-1}$. Vindesine was reduced by 50% if WHO grade II peripheral neurotoxicity was encountered and omitted if WHO grade III or IV toxicity occurred. Patients who experienced other types of grade III non-haemotological toxicity had both their gemcitabine and vindesine dose reduced by 50%, and patients with grade IV non-haematological toxicity were taken off study unless a response to chemotherapy had occurred. In these latter cases, a 50% dose reduction could be applied when toxicity had disappeared at the discretion of the physician in charge of treatment.

A dose escalation of vindesine and gemcitabine of 20% was permitted if the previous course was associated with a WHO toxicity score $\leq$ 1.

### Quality-of-life evaluation

Quality-of-life was assessed by the European Organization for Research and Treatment of Cancer (EORTC) Core Quality-of-Life Questionnaire, the QLQ-C30 (version 1), supplemented by a lung cancer-specific questionnaire module, the QLQ-LC13. Both the EORTC QLQ-C30 and the QLQ-LC13 have been thoroughly pretested in large samples of lung cancer patients (Aaronson et al, 1993; Bergman et al, 1994), and they have demonstrated good reliability and validity and sufficient ability to detect changes in patients' clinical status over time. In addition, two test items hypothesized to measure aspects of peripheral neuropathy were employed.

For all symptom measures and the majority of function measures, response categories ranged from 'not at all', scored as 1, to 'very much', scored as 4. The response categories for items referring to physical and role function were dichotomous (yes or no), while overall quality of life was scored on a semilinear scale, ranging from 1 (very poor) to 7 (excellent). Scale scores were calculated by dividing the sum of scores from individual items by the number of items. For analysis and presentation, all scores (both single item and scale scores) were linearly transformed to a 1–100 scale. For function scales and overall quality of life, 100 means best possible reported function (i.e. complete absence of dysfunction), whereas for the remaining scales and items measuring symptoms and side-effects 100 means worst possible reported complaints.

*© Cancer Research Campaign 1999*

All patients were expected to complete the quality-of-life questionnaire at pretreatment, after 4, 8 and 16 weeks and, if treatment was continued for further courses, after every second following treatment course. In addition, quality-of-life assessment was carried out at the time of treatment discontinuation.

### Statistics

The relation between clinical variables and quality-of-life measures was tested by factorial ANOVA or Spearman's non-parametric correlation analysis. Change in quality-of-life measures over time was analysed by repeated-measures ANOVA. A stepwise multiple regression analysis was carried out to evaluate the extent to which symptoms and side-effects influenced the variance in overall quality-of-life ratings during treatment. In order to reduce the random effects of multiple comparisons, the level of statistical significance was set to $P \leq 0.01$.

### RESULTS

### Patients and treatment duration

Between August 1992 and June 1994, 42 patients entered the study, ten in Sweden and 32 in Denmark. Patient characteristics are listed in Table 1. The median age was 56 years (range 37–70). Two patients with stage III A disease were inoperable because of mediastinal lymphadenopathy. Of the 42 patients entered into the phase II study and evaluated for toxicity, only 40 were valuable for response. One patient was excluded before the completion of one treatment course because of early death due to pulmonary embolism and one because of lack of evaluable disease.

The number of completed treatment courses are given in Table 2. The 42 patients received a total of 168 treatment courses (one treatment course equals 4 weeks) of the gemcitabine and vindesine combination. The median number of completed treatment courses was 3 (range 0–19) and median treatment duration was 14 weeks (range 2–29). Ten patients (23%) received six or more treatments.

**Table 1** Patients characteristics ($n$ = 42)

| | *n* | % |
|---|---|---|
| Treatment centre | | |
| Rigshospitalet | 14 | (33%) |
| Herlev Hospital | 18 | (43%) |
| Renströmska Hospital | 10 | (24%) |
| Gender | | |
| Male | 24 | (57%) |
| Female | 18 | (43%) |
| Histology | | |
| Squamous cell | 13 | (31%) |
| Adenocarcinoma | 22 | (52%) |
| Large-cell carcinoma | 7 | (17%) |
| Tumour grade | | |
| Well differentiated | 2 | (5%) |
| Moderately differentiated | 3 | (7%) |
| Poorly differentiated | 19 | (45%) |
| Unknown | 18 | (43%) |
| Stage | | |
| III A | 2 | (5%) |
| III B | 17 | (40%) |
| IV | 23 | (55%) |
| Performance status (WHO) | | |
| 0 | 4 | (10%) |
| 1 | 35 | (83%) |
| 2 | 3 | (7%) |

**Table 2** Number of completed treatment courses (one cycle = 4 weeks)

| No. courses | No. patients | % |
|---|---|---|
| 0 | 1 | 2.4 |
| 1 | 5 | 11.9 |
| 2 | 6 | 14.3 |
| 3 | 11 | 26.2 |
| 4 | 7 | 16.7 |
| 5 | 2 | 4.8 |
| 6 | 4 | 9.5 |
| 7 | 3 | 7.1 |
| 8 | 2 | 4.8 |
| 19 | 1 | 2.4 |

Median three completed courses (range 0–19).

### Toxicity

The haematological as well as the non-haematological toxicity as indicated by laboratory measurement are depicted in Table 3. WHO grade III–IV leucocytopenia was observed in five patients (12%). Neutrophil counts were available in 33 patients, of whom 13 (39%) had grade IV neutropenia. There were no septic deaths. Thrombocytopenia grade III–IV was observed in six patients (15%) without any bleeding episodes.

The liver toxicity is outlined in Table 3. A transient rise in alkaline phosphatase equal to WHO grade I–III toxicity occurred in 25 patients (60%) and a rise in alanine transaminase equal to WHO grade I–III occurred in 33 patients (79%). One patient (2%) had an increase in bilirubin equal to WHO grade IV toxicity but no other grade IV liver toxicity was observed. The hepatic toxicity was reversible in all cases.

With respect to renal toxicity, four patients (13%) exhibited a decrease in glomerular filtration rate below normal limits as indicated by chrom-EDTA clearance. This was reversible in three patients, while one patient developed a non-reversible renal insufficiency. Two patients had an increase in creatinine level to WHO grade I or III level (Table 3). Proteinuria was observed in 28 patients (67%) and haematuria in 24 patients (57%) (Table 4). The mechanism for the renal toxicity is unknown.

A variety of non-laboratory toxicities were encountered, as indicated in Table 4. The majority of patients (34, 81%) developed peripheral neuropathy, with nine patients (21%) experiencing WHO grade III neuropathy and none grade IV neuropathy (Table 4).

Ten patients (24%) complained of dyspnoea (Table 4). The ventilation capacity was measured by peak-flow measurements immediately before chemotherapy, immediately after chemotherapy and 1 and 2 h later. This procedure was initiated after nine patients had been included in the study because one patient developed severe dyspnoea causing hospitalization. A peak-flow reduction of more than 50% within 2 h after drug infusion compared with preinfusion level occurred in three patients (9%) and the dyspnoea caused hospitalization in two patients but was uniformly reversible. In one of these patients the dyspnoea disappeared spontaneously within half an hour, whereas the other patient developed respiratory insufficiency with dyspnoea and cyanosis. On auscultation there was a

© Cancer Research Campaign 1999

**Table 3** Worst haematological and non-haematological toxicity during gemcitabine and vindesine treatment in 42 patients

| | WHO grades [no. of patients (%)] | | | | |
|---|---|---|---|---|---|
| **Toxicity** | **0** | **1** | **2** | **3** | **4** |
| Haematological | | | | | |
| Leucocyte count | 8 (19) | 15 (36) | 14 (33) | 4 (10) | 1 (2) |
| Neutrophil count[a] | 9 (27) | 7 (21) | 4 (12) | 10 (30) | 3 (9) |
| Thrombocyte count | 31 (74) | 2 (5) | 3 (7) | 4 (10) | 2 (5) |
| Haemoglobin | 15 (36) | 17 (41) | 9 (21) | 1 (2) | 0 |
| Non haematological | | | | | |
| Alkaline phosphatase | 17 (41) | 16 (38) | 7 (17) | 2 (5) | 0 |
| Alanine transaminase | 9 (21) | 10 (24) | 15 (36) | 8 (19) | 0 |
| Bilirubin | 39 (93) | 2 (5) | 0 | 0 | 1 (2) |
| Creatinine | 40 (95) | 1 (2) | 0 | 1 (2) | 0 |

[a]Data on neutrophil counts available in 33 patients.

**Table 4** Toxicity observed in 42 patients treated with gemcitabine and vindesine

| | WHO grades [no. of patients (%)] | | | | |
|---|---|---|---|---|---|
| **Toxicity** | **0** | **1** | **2** | **3** | **4** |
| Nausea/vomiting | 10 (24) | 12 (29) | 19 (45) | 1 (2) | 0 |
| Alopecia | 23 (55) | 4 (10) | 10 (24) | 5 (12) | 0 |
| Infection | 32 (76) | 6 (14) | 4 (10) | 0 | 0 |
| Fever | 27 (64) | 7 (17) | 8 (19) | 0 | 0 |
| Cutaneous (rash) | 26 (62) | 5 (12) | 11 (26) | 0 | 0 |
| Pulmonary (dyspnoea) | 32 (76) | 6 (14) | 1 (2) | 2 (5) | 1 (2) |
| Proteinuria | 14 (33) | 25 (60) | 3 (7) | 0 | 0 |
| Haematuria | 18 (43) | 22 (52) | 0 | 2 (5) | 0 |
| Diarrhoea | 36 (86) | 3 (7) | 3 (7) | 0 | 0 |
| Constipation | 24 (57) | 13 (31) | 2 (5) | 3 (7) | 0 |
| Peripheral neuropathy | 8 (19) | 9 (21) | 16 (38) | 9 (21) | 0 |

prolonged expiration phase without crepitation and an arterial puncture showed hypoxia and hypercapnia. ECG showed sinus rhythm and chest radiography revealed no signs of new events. Both acute myocardial infarction and pulmonary embolism were ruled out. The patients were treated with inhalation of terbutaline and intravenous administration of loop diuretics, terbutaline, theophyllamine and glucocorticoids. The pulmonary symptoms were completely reversible and the second patient was not re-exposed to gemcitabine and vindesine.

The cumulated median administered doses of gemcitabine and vindesine were 9000 mg $m^{-2}$ (range 1000–48 750) and 21 mg $m^{-2}$ (range 3–45) respectively. The cumulated dose intensity (planned dose divided by actual administered dose) was 84% for gemcitabine and 70% for vindesine. The reasons for dose reductions are outlined in Table 5. The most frequent reasons for vindesine dose reduction were paraesthesia (19 patients) and leucopenia (nine patients).

### Responses

The objective response rate for 40 assessable patients was 20% (95% confidence limits 9–36%), with one complete response (2.5%) and seven partial responses (17.5%). Stable disease was noted in 28 patients (70%) and four patients (10%) had disease progression. The median response duration was 31 weeks (range 11–83 weeks).

The median overall survival time from start of treatment was 31 weeks (range 2–171 weeks), and the 1-and 2-year survival rates were 33% and 7% respectively. All patients have since died.

### Quality of Life

Forty-one patients were evaluable for quality of life at baseline (pretreatment). Follow-up (on-treatment) assessments after 4, 8 and 12–16 weeks were completed by 33, 26 and 22 patients respectively. Overall compliance in terms of completed/expected questionnaires was 73%.

Function and global quality-of-life scores at baseline are displayed in Table 6. For comparison, the baseline scores obtained in a large international field study of the EORTC questionnaire (Aaronson et al, 1993), comprising a comparable group of patients with non-resectable lung cancer, are included in the table. At baseline, women reported worse emotional function than did men (emotional function score 58 in women vs. 76 in men, $P = 0.001$). No other significant interaction effects of pretreatment objective clinical variables on patient-rated function measures were observed (Table 1).

Figure 1 displays the mean scores for physical, emotional and social function and overall quality of life during the first four treatment courses. Following the second treatment course, there was a significant deterioration of the mean self-reported physical function ($P < 0.0001$). A similar trend towards deterioration was seen in social function ($P < 0.05$). In contrast, the mean emotional function score improved after one treatment course ($P < 0.01$), although the mean overall quality-of-life score remained essentially unchanged during treatment. The deterioration of physical function during the first two treatment cycles was most pronounced in patients who did not have an objective tumour

 © Cancer Research Campaign 1999

**Table 5** Reasons for dose reductions[a] (cumulated) among 42 evaluable patients

| | No. of patients (%) | |
|---|---|---|
| | **Gemcitabine** | **Vindesine** |
| Leucopenia | 18 | 9 |
| Thrombocytopenia | 11 | 1 |
| Paraesthesiae | | 19 |
| Constipation | | 5 |
| Oedema | 3 | |
| Haematuria | 1 | 2 |
| Renal function | 2 | |
| SGOT elevation | 1 | |
| Rash | | 1 |
| Dyspnoea | | 1 |

[a]Individual patients may have more than one reason for dose reduction.

**Table 6** Baseline (pretreatment) function and overall quality-of-life scores in 41 study patients. For comparison, reference baseline data on 305 patients with non-resectable lung cancer and similar performance status (Aaronson et al 1993) are included

| | **Gemcitabin/vindesine data mean (s.d.)** | **EORTC data mean (s.e.)** |
|---|---|---|
| Function scale | | |
| Physical | 67.5 (24.3) | 65.8 (1.6) |
| Role | 59.5 (37.0) | 57.3 (2.2) |
| Emotional | 65.5 (22.5) | 70.0 (1.3) |
| Cognitive | 85.3 (20.5) | 83.6 (1.2) |
| Social | 82.2 (21.2) | 77.3 (1.6) |
| Overall quality of life | 55.2 (23.5) | 56.7 (1.3) |

response ($P$ = 0.033 for interaction effect; two-factor repeated measures ANOVA).

Mean symptom scores at baseline and changes to follow-up assessments during treatment are displayed in Table 7. There was a significant increase (i.e. deterioration) in the mean fatigue score after two treatment courses ($P < 0.0001$), although the deterioration did not proceed in patients who received further treatment. Increased levels of constipation were reported after the first treatment course ($P < 0.01$). Patients' ratings of hair loss and symptoms related to peripheral neuropathy increased significantly during treatment ($P < 0.0001$ at all follow-up assessments). The remaining symptom score changes were non-significant.

The extent to which disease- and treatment-related symptoms influenced the overall quality-of-life ratings during treatment was evaluated by means of a stepwise regression analysis. The regression model included pain, fatigue, dyspnoea and appetite loss as independent variables because the variables correlated with quality of life in univariate analysis (data now shown). After 4 weeks, pain and fatigue entered the multivariate model, explaining 66% of the variance of overall quality-of-life ratings (multiple $R$ = 0.814), whereas after 8 weeks only fatigue entered the model.

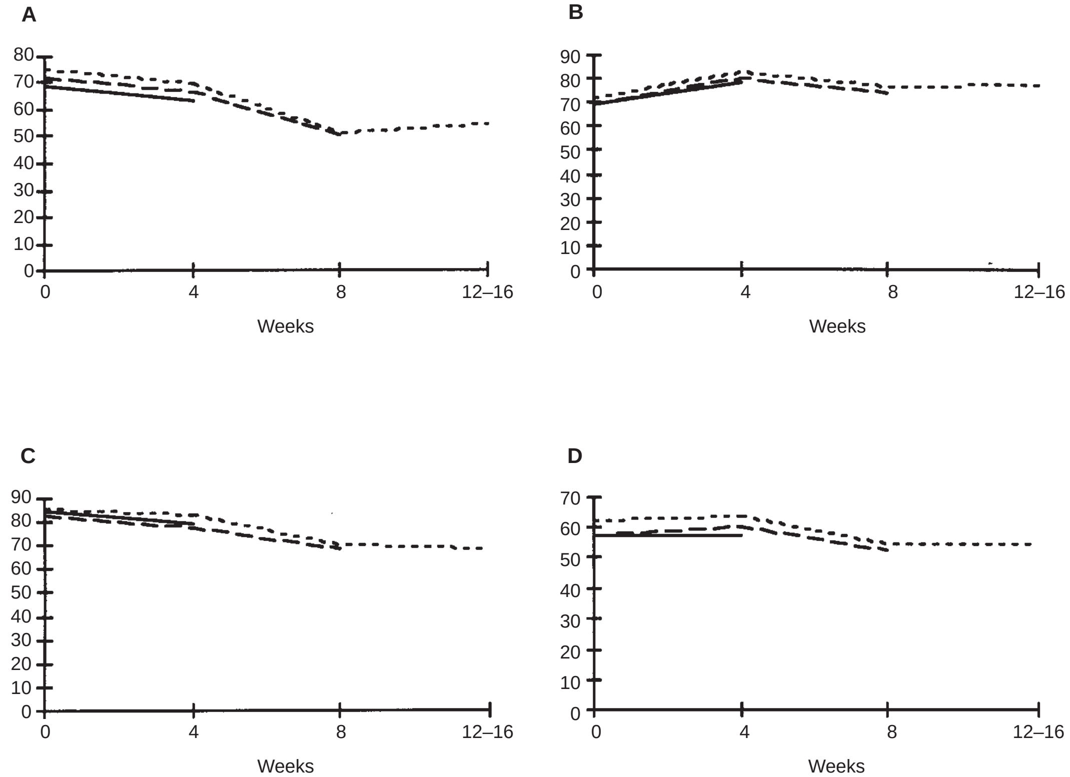

**Figure 1** Changes in mean physical (**A**), emotional (**B**) and social function (**C**) and quality-of-life (**D**) scores during treatment with gemcitabin and vindesine. Score range from 0 to 100, with 100 indicating the best possible rating (i.e. absence of reported dysfunction). Line charts are presented separately for patients measured from start of treatment to week 4 (–––, $n$ = 33), from start of treatment to week 8 (— —, $n$ = 26) and from start of treatment to weeks 12–16 (– – –, $n$ = 22)

**Table 7** Baseline (pretreatment) symptom scores in 41 evaluable patients and mean score changes from baseline during treatment. Positive score changes indicate increased symptoms (i.e. deterioration); negative score changes indicate decreased symptoms (i.e. improvement)

| | Mean score (s.d.) at baseline (*n* = 41) | Mean score changes from baseline | | |
|---|---|---|---|---|
| | | 4 weeks (*n* = 34) | 8 weeks (*n* = 27) | 12–16 weeks (*n* = 22) |
| Pain | 34.5 (30.9) | 1.6 | 3.5 | 13.1 |
| Fatigue | 42.5 (23.6) | 11.2 | 21.5 | 13.8 |
| Dyspnoea | 30.6 (23.6) | 1.3 | 7.2 | 6.7 |
| Sleep disturbance | 27.3 (29.5) | –4.6 | –1.1 | 4.5 |
| Appetite loss | 36.5 (33.7) | 5.1 | 10.0 | 9.4 |
| Nausea/vomiting | 15.5 (24.8) | 2.2 | –2.4 | –5.5 |
| Coughing | 46.9 (29.7) | –11.3 | –12.5 | –11.7 |
| Haemoptysis | 4.9 (14.0) | 0 | –3.8 | –4.3 |
| Constipation | 10.5 (20.2) | 18.8 | 13.6 | 8.7 |
| Diarrhoea | 7.1 (15.6) | 3.1 | 5.0 | –2.9 |
| Peripheral neuropathy | 7.6 (8.1) | 28.3 | 49.4 | 53.0 |
| Hair loss | 0 | 38.4 | 54.4 | 46.3 |

### DISCUSSION

The goal of combination chemotherapy is to increase treatment efficiency through the synergistic combination of drugs, thus avoiding cross-toxicities. Usually, for an additive effect to be exerted, the cytostatic agents have to possess different modes of action on the neoplastic cells. Although the latter point is fulfilled with the current combination of gemcitabine and vindesine, the first point is not completely satisfied. Because of the partly overlapping toxicity, especially with respect to myelotoxicity, neither of the two cytostatic agents applied could be used in the optimal dose from the studies of single-agent doses. The response rate of 20% observed with gemcitabine and vindesine with the currently applied doses was disappointing as it was of the same order as achieved with either gemcitabine (Hansen et al, 1997) or vindesine alone (Sørensen et al, 1993). However, with the present haematological toxicity data at hand, and with 39% of the patients having neutropenia WHO grade III–IV (Table 3), it does not appear feasible to increase further the dosage of the individual agents used in this combination.

Renal toxicity was more pronounced than would be expected based on the toxicity profile of both gemcitabine and vindesine. In four patients (13%) the glomerular filtration rate was decreases below normal limits as indicated by chrom-EDTA clearance; this effect was totally reversible in three patients but progressed to renal insufficiency in one patient. Also, the occurrence of proteinuria in 28 patients (67%) and haematuria in 24 patients (57%) indicates that this antineoplastic regimen can be attributed some renal toxicity, the precise mechanism for which is as yet undetermined. One might speculate whether it may be a result of pharmacokinetic changes when these two drugs are given together. This point has, however, not been investigated.

Also, the mechanism for the dyspnoea observed in some patients remained unknown. However, based on the clinical appearance and the responsiveness to bronchodilatating drugs points towards an element of bronchoconstriction, though a minor pulmonary oedema might also, theoretically, contribute to the condition.

The principal components of health-related quality of life may be affected to various degrees, and in different directions, by cancer and its treatment. Thus, a multidimensional approach to quality-of-life assessment enhances the sensitivity in detecting differentiated associations between disease or treatment and subjective outcomes. This was clearly demonstrated in the present study, in which patients with advanced NSCLC deteriorated with regard to physical functioning and fatigue, but showed improved emotional functioning, and retained their overall quality of life during treatment. The pattern of changes is consistent with previous findings in both small-cell and non-small-cell lung cancer populations receiving chemotherapy (Kaasa et al, 1988; Bergman et al, 1992; Aaronson et al, 1993), although the magnitude of changes sometimes diverged from what could be expected. In particular, the increase in fatigue levels during treatment was more pronounced than has been reported in clinical trials of some other chemotherapy regimens with moderate activity in NSCLC, e.g. carboplatin/etoposide (Helsing et al, 1998).

Tumour response is, to some extent, associated with changes in subjective outcome measures during treatment for lung cancer, and the lack of anti-tumour activity has been associated with a deterioration of functional measures (Bergman et al, 1991; Kaasa and Masthekaasa, 1998). In the present study, the mean physical function value dropped earlier in non-responding patients than in responders, supporting the hypothesis that objective tumour response may contribute to a delay in the functional deterioration that will eventually occur in a vast majority of patients with non-resectable lung cancer.

When interpreting the variation in quality-of-life dimensions during treatment, one has to consider the marginals for changes from baseline measurements. Patient-rated quality of life and physical functioning, as measured with the EORTC questionnaire, have been shown to vary significantly from observed-rated performance status (Aaronson et al, 1993). In the present study, 91% of the patients had a WHO performance status of 0 or 1 at baseline, indicating that they had no major functional limitations prior to the onset of treatment. Consequently, the marginals for functional improvement during treatment were small, and the on-treatment quality-of-life assessments were likely to capture principally adverse treatment effects and symptoms due to disease progression. Taking this view, a delay in deterioration of quality of life may be interpreted as a beneficial effect of treatment on the patients' well-being.

In order to improve further the activity for the new chemotherapeutic agents with significant activity in NSCLC, they most likely are to be combined with a platinum analogue, which has been the focus of several investigations (Sørensen, 1994; Cojean et al, 1995; Edelman et al, 1996). A review of vindesine in NSCLC covering

*© Cancer Research Campaign 1999*

accumulated data from eight randomized studies, including a total of 466 patients receiving vindesine + cisplatin, revealed 141 responding patients (30% response rate, 95% confidence limits 26–34%), with response rate in the individual studies ranging from 19% to 67% (Sørensen et al, 1993). Similarly, gemcitabine has been evaluated in combination with cisplatin and in two dose escalation studies involving a total of 22 patients, 13 responses were noted with response rates in the individual studies ranging from 58% (Sheperd et al, 1994) to 60% (Steward et al, 1994). Similarly, two phase II studies using this combination observed responses in 26 out of 48 patients (54%, 95% confidence limits 39–69%; Crino et al, 1997), and in 26 out of 50 evaluable patients (52%, 95% confidence limits 37–66%; Abratt et al, 1997). Whether these promising response rates will persist when more patients are treated with this combination in randomized trials is awaited with interest, as is the effect on survival and symptomatology score.

A dose-escalation study evaluating a combination of vinorelbine and gemcitabine revealed a preliminary response rate of 20% (95% confidence limits 8–39%), but the maximal tolerated dose was not reached (Krajnik et al, 1997) Further evaluation of the active single agents in NSCLC in combination with a platinum analogue with or without additional cytostatic agents included, should have priority in order to further enhance our possibilities of influencing the natural course of this grave disease.

With respect to quality of life, treatment with gemcitabine and vindesine in non-resectable NSCLC did not significantly interfere with patients' emotional function or overall quality of life as measured by the EORTC QLQ-C30, but it produced significant levels of peripheral neurotoxic symptoms, and it may have contributed to an increase in fatigue levels that was a significant determinator of overall quality-of-life ratings during treatment. In view of a modest objective tumour response rate, the subjective treatment outcomes do not support gemcitabine and vindesine used in the current schedule as a combination regimen of choice for further comparative chemotherapy trials.

### ACKNOWLEDGEMENT

This study was supported by an educational grant from Eli Lilly Company.

### REFERENCES

Aaronson NK, Ahmedzai S, Bergman B et al (1993) The European Organization for Research and Treatment of Cancer QLQ-C30: a quality-of-life instrument for use in international clinical trials in oncology. *J Natl Cancer Inst* **85**: 365–376

Abratt RP, Bezwoda WR, Goedhals L and Hacking DJ (1997) Weekly gemcitabine with monthly cisplatin: effective chemotherapy for advanced non-small-cell lung cancer. *J Clin Oncol* **15**: 744–749

Anderson H, Thatcher N, Walling J et al (1994) Gemcitabine and palliation of symptoms in non-small cell lung cancer (NSCLC). *Proc* **13**: 367

Bakker W, van Oosterom AT and Aaronson NK (1986) Vindesine, cisplatin, and bleomycin combination chemotherapy in non-small cell lung cancer: survival and quality of life. *Eur J Cancer Clin Oncol* **22**: 963–970

Batel-Copel LM, Kornblith AB, Batel PCCC and Holland JC (1997) Do oncologists have an increasing interest in the quality of life of their patients? A literature review of the last 15 years. *Eur J Cancer* **33**: 29–32

Bayssas M, Gouveia J, de Vassal F, Misset J-L, Schwarzenberg L, Ribaus P, Musset M, Jasmin C, Hayat M and Mathe G (1980) Vindesine: a new vinca alkaloid. *Recent Res Cancer Res* **74**: 91–97

Bergman B, Sullivan M & Sörenson S (1991) Quality of life during chemotherapy for small cell lung cancer. I. An evaluation with generic health measures. *Acta Oncol* **30**: 947–957

Bergman B, Sullivan M and Sörenson S (1992) Quality of life during chemotherapy for small cell lung cancer II. A longitudinal study of the EORTC Core Quality of Life Questionnaire and Comparison with the Sickness Impact Profile. *Acta Oncol* **31**: 19–28

Bergman B, Aaronson NK, Ahmedzai S et al (1994) The EORTC QLQ-L13: a modular supplement to the EORTC Core Quality of Life Questionnaire (QLQQ-C30) for use in lung cancer clinical trials. *Eur J Cancer* **30A**: 635–642

Billingham LJ, Cullen MH, Woods J et al (1997) Mitomycin, ifosfamide and cisplatin (MIC) in non-small cell lung cancer: results of a randomised trial evaluating palliation & quality of life. *Lung Cancer* **18** (suppl.1): 9–10

Cojean I and Le Chevalier T (1995) Chemotherapy of stage IIIB and IV non-small-cell lung cancer. *Ann Oncol* **6** (suppl.3): S41–S44

Comis RL and Friedland DM (1995) New chemotherapy agents in the treatment of advanced non-small cell lung cancer: an update including data from the Seventh World Conference on Lung Cancer. *Lung Cancer* **12** (suppl. 2): S63–S99

Crino L, Scagliotti G, Marangolo M et al (1997) Cisplatin-Gemcitabine Combination in Advanced Non-Small-Cell Lung Cancer: A Phase II study. *J Clin Oncol* **15**: 297–303

Edelman MJ and Gandara DR (1996) Promising new agents in the treatment of non-small cell lung cancer. *Cancer Chemother Pharmacol* **37**: 385–393

Ganz PA, Figlin RA, Haskell CM et al (1989) Supportive care versus supportive care and combination chemotherapy in metastatic non-small cell lung. Does chemotherapy make a difference? *Cancer* **63**: 1271–1278

Groth S and Aasted M (1981) $^{51}$Cr-EDTA clearance determined by one plasma sample. *Clin Physiol* **1**: 417–425

Guchelaar H-J, Richel DJ and van Knapen A (1996) Clinical, toxicological and pharmacological aspects of Gemcitabine. *Cancer Treat Rev* **22**: 15–31

Hansen HH and Sørensen JB (1997) Efficacy of single-agent gemcitabine in advanced non-small cell lung cancer: a review. *Sem Oncol* **24** (suppl. 2): S38–S41

Helsing M, Bergman B, Thaning L and Hero U (1998) Quality of life and survival in patients with advanced non-small cell lung cancer receiving supportive care plus chemotherapy with carboplatin and etoposide or supportive care only. A multicentre randomized phase III trial. *Eur J Cancer* (in press)

Kaasa S and Mastekaasa A (1988) Psychosocial well-being of patients with inoperable non-small cell lung cancer. *Acta Oncol* **27**: 829–835

Kaasa S, Mastekaasa A and Thorud E (1988) Toxicity, physical function and everyday activity reported by patients with inoperable non-small lung cancer in a randomised trial (chemotherapy versus radiotherapy). *Acta Oncol* **27**: 343–349

Kosty MP, Fleishman SB, Herndon JE et al (1994) Cisplatin, vinblastine, and hydrazine sulfate in advanced, non-small cell lung cancer: a randomized placebo-controlled, double-blind phase III study of Cancer and Leukemia Group B. *J Clin Oncol* **12**: 1113–1120

Krajnik G, Mohn-staudner A, Marhold F et al (1997) Vinorelbine/gemcitabine in advanced non-small cell lung cancer (NSCLC): a phase I trial. *Proc Am Soc* **16**: 206

Maasilta PK, Rautonen JK, Mattson MT and Mattson KV (1990) Quality of life assessment during chemotherapy for non-small cell lung cancer. *Eur J Cancer* **26**: 706–708

Minet P, Bartsch P, Chevalier P et al (1987) Quality of life in patients with non-small cell lung cancer treated with chemotherapy. *Radiother Oncol* **8**: 217–230

Pujol JL, Monnier A, Berille et al (1994) Phase II study of nitrosourea fotemustine as single-drug chemotherapy in poor-prognosis non-small cell lung cancer. *Br J Cancer* **69**: 1136–1140

Sarna LP (1990) Impact of chemotherapy on the quality of life and functional status of older adults with non-small cell lung cancer. *Dis Abst Int* **51**: 1748

Schipper H, Clinch J, McMurray A and Levitt M (1984) Measuring the quality of life in cancer patients: The Functional Living Index-Cancer. *J Clin Oncol* **2**: 472–483

Shepherd F, Cormier Y, Burkes R et al (1994) Phase I study of gemcitabine and cisplatin for advanced non-small cell lung cancer. *Lung Cancer* **11** (suppl.): 116

Sørensen JB (1994) The role of chemotherapy in non-small cell lung cancer. *Radiol Oncol* **28**: 386–394

Sørensen JB and Hansen HH (1993) Is there a role for vindesine in the treatment of non-small cell lung cancer? *Invest New Drugs* **11**: 103–133

Souquet PJ, Chauvin F, Boissel JP, Cellerino R, Cormeier Y, Ganz PA, Kaasa S, Pater JL, Quoix E, Rapp E, Tumarello D, Williams J, Woods BL and Bernard JP (1993) Polychemotherapy in advanced non-small cell lung cancer: a meta-analysis. *Lancet* **342**: 19–21

Steward WP, Dunlop DJ, Cameron C et al (1994) Cisplatin + gemcitabine in non-small cell lung cancer. A phase I dose escalation study. *Lung Cancer* **11** (suppl.): 114

WHO (1979) *Handbook for Reporting Results of Cancer Treatment*, Offset publication 42, World Wealth Organization: Geneva

© Cancer Research Campaign 1999
